# The Influence of Inhibition of Return and Level-Priming on the Global Precedence Effect

**DOI:** 10.1155/2020/4817901

**Published:** 2020-09-01

**Authors:** Yan Wu, Qi Li, Yuanzi Liu

**Affiliations:** Changchun University of Science and Technology, Changchun, Jilin, China

## Abstract

The aim of the reported experiment was to investigate the effects of inhibition of return (IOR) and level-priming on the global precedence effect (GPE). The classical hierarchical stimuli combined with IOR and the level-priming paradigm were used. The participants selectively attended to the global or local features of compound numerals. The results showed that IOR inhibited the response to the global and local features; moreover, the inhibition effect on the perception of the global features was stronger than that of the local features in the stage of inhibitory processing, resulting in the disappearance of GPE. However, level-priming promoted the response to global and local features, and the promotion effect was stronger on local features, leading to the disappearance of GPE as well. These findings suggested that hierarchical processing was affected by IOR and level-priming, which were correlated with selective attention. Thus, it indicated that global precedence could be involved in attentional mechanisms.

## 1. Introduction

In the recent years, several studies have reported that participants always showed shorter reaction times (RTs) to features at the global level (elements in the global position) than to features at the local level (elements in the local position) for a compound stimulus (a large element composed of many small elements) [1–3]. In 1977, Navon set up a global/local task [4]. In this task, participants were presented with some compound letter stimuli (a large letter composed of many small letters). There were two conditions: the congruent condition (e.g., a larger letter “A” composed of many small “A”s) and the incongruent condition (e.g., a larger letter “A” composed of many small “S”s). The results showed that RTs to identify the global letters were shorter than the RTs to identify the local letters (the global RT advantage effect), and interference from the global letters when participants identified the local letters was larger than in the opposite case (the global-to-local interference effect). These two effects have been referred to as the global precedence effect (GPE) in the literature. The global RT advantage exists in the processing of early perception, and it can also be mediated by early perceptual processes [5–7]. Furthermore, works by Han, Liu, Yund, and Woods [8] and Han, He, and Woods [9] showed that modulation of brain potentials by hierarchical processing occurred at the early sensory-perceptual levels where spatial selective attention also operated.

It is well known that inhibition of return (IOR) has an effect on spatial selective attention; when a cue is used to predict the location of a forthcoming target, subjects respond faster and with greater accuracy to targets in the cued location than to targets presented in noncued locations [10]. However, when the time interval between the cue and the target stimulus (i.e., the stimulus onset asynchrony (SOA)) is longer than 300 ms, responses to targets in cued locations are slower and less accurate than are responses to targets presented in noncued locations. This effect has been termed IOR [11]. The amplitude of EEG signals related to early perceptual activities in the cued location was obviously reduced compared with the noncued location. Thus, IOR comes from the change of early perceptual processes to a certain extent. This change was manifested as a significant inhibition effect on the cued locations in the response of participants [12–15]. Whether the GPE will be affected by the mechanism of IOR which has a close relationship with the spatial attention processes is worth studying. The first purpose in this study was to investigate whether IOR has a significant effect on the GPE.

The study explores whether there is any connection between the mechanism of GPE and level-priming that is also closely related to early perceptual processes [16, 17] and whether the GPE can be mediated by level-priming. In 1982, Ward conducted an experiment wherein he presented stimuli at the global level or local level. In the experiment, participants identified the stimuli in pairs of trials in a particular sequence (global then global, global then local, local then global, and local then local). In the block of repeated level (the stimuli were sequentially presented at the same level), response times (RTs) were faster than those in the changed level (the stimuli were sequentially presented at different levels). This so-called level-priming was found to affect whether participants identified local or global stimuli. Level-priming is an automatic attentional persistence to the perceptual scale. This highlights flexibility in the perceptual system; when confronted with repeated-level stimuli, perceivers make use of relative information obtained previously in the early perceptual processes to guide subsequent selection in the global/local tasks [16, 18]. Investigating whether this previous information will have some influence on the GPE, which is also closely related to the early perceptual process, is the second purpose of this research.

The present research applied the paradigm of IOR and level-priming to global/local tasks. We used two stimuli which were presented in a sequence in the experiment. The first stimulus in a trial was designed to explore the influence of IOR on the GPE. If IOR has an inhibitory effect on the GPE, there will be no significant GPE in the processing of the first stimulus presented in the inhibition locations. The second stimulus was mainly to study the influence of level-priming on the GPE. If level-priming had a significant effect on the GPE, the GPE would change during the processing of the second stimulus in the repeated-level stimuli condition.

## 2. Methods and Materials

### 2.1. Participants

Eighteen healthy volunteers (nine males and nine females aged 20–28 years) participated in the present study. All participants had normal or corrected-to-normal vision, and all were right-handed. All provided written informed consent. The experimental protocol was approved by the Ethics Committee of Changchun University of Science and Technology.

### 2.2. Stimuli

**Figure 1:**
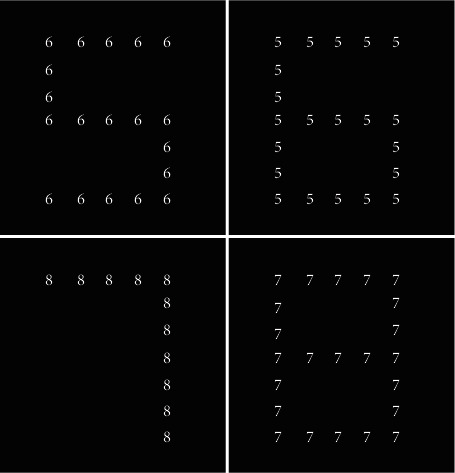
Compound stimulus patterns used in the experiment. Top: compound stimuli with numerals 5 and 6, respectively. Bottom: compound stimuli with numerals 7 and 8, respectively.

Stimuli were presented on a 19-inch Samsung cathode ray tube monitor positioned 60 cm from the subject's head. Responses were recorded through software “Presentation”. All compound stimuli in this study were composed of the following numerals: 5, 6, 7, and 8. Each compound stimulus had two distinct feature levels (global: large numeral; local: small numeral). The small numeral (0.9 ° × 1.1 °) was used to create the large numeral (6.4 ° × 8.0 °) based on a 5 × 7 matrix. Four different patterns of these numerals were used to create compound stimuli, in which the global and the local numerals differed. That is, small numerals 5 and 7 formed large numerals 6 and 8, respectively, and vice versa (see Figure 1).

### 2.3. Procedure

**Figure 2:**
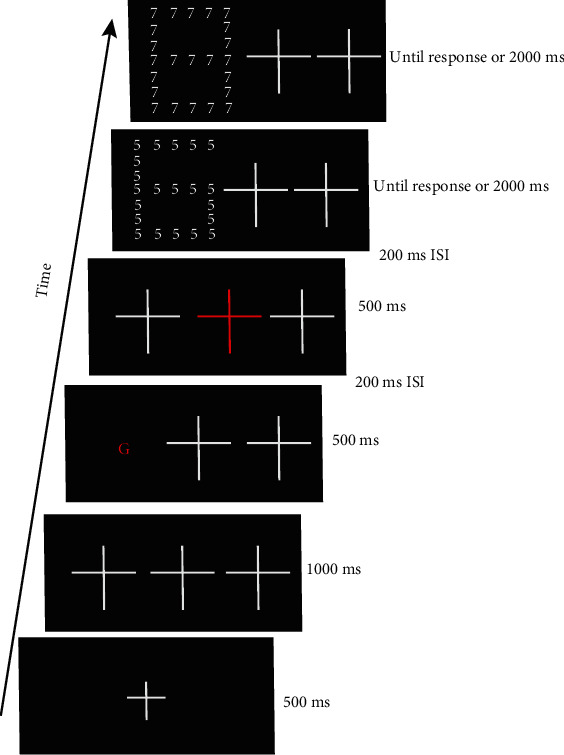
Experimental sequence: each trial began with a fixation cross “+”, and after 500 ms, the fixation cross was replaced by three larger crosses for 1000 ms. Then, one of the peripheral crosses was replaced by a cue “G” or “L” in red for 500 ms. The red cue “G” indicated that the target of the first stimulus display was the global numeral, while the red cue “L” indicated that the target of the first display was the local numeral. After an ISI of 200 ms, the peripheral crosses were presented again, and the central cross turned red for 500 ms. This was followed by a further ISI of 200 ms, and the first and second displays were presented. In the figure, it is an example to illustrate the experimental process.

Participants were seated 60 cm from the computer screen, after which the experimenter explained the task orally.  Figure 2 shows the procedure and duration of the stimuli employed in the experiment. Each trial began with a fixation cross “+” (3.5 ° × 4.2 °) presented in the middle of the screen. After 500 ms, the fixation cross was replaced by three larger crosses (6.9 ° × 8.0 °). These crosses were arranged horizontally, with one in the center being flanked by two others on the periphery. The distance from each peripheral cross to the middle cross was 4.1°. After 1000 ms, one of the peripheral crosses was replaced by a cue “G” or “L” in red (2.4 ° × 2.9 °) for 500 ms. After an interstimulus interval (ISI) of 200 ms, the peripheral crosses were presented again, and the central cross turned red for 500 ms. This was followed by a further ISI of 200 ms before the first and second displays were presented. This central red cross was used to help participants reorient their attention to the center of the screen before the patterns were presented, which was necessary to observe the IOR effects. The compound stimuli were, then, randomly presented to the left or right of the central fixation, and participants had 2000 ms to respond. If no response was given during that time, the trial was registered as an error and the next trial was initiated.

The experiment consisted of two blocks (changed- and repeated-level conditions) of 200 experimental trials and 20 practice trials each (trials in each block were divided into five sessions consisting of 40 experimental trials and 4 practice trials). Each block consisted of attended-left and attended-right conditions of 100 experimental trials and 10 practice trials each. In each block, half the trials used global numerals as target stimuli and the other half used local numerals as targets. The cues were valid in 150 experimental trials (15 practice trials) and invalid in 50 trials (5 practice trials) in each block. Each session was followed by a 5-minute rest interval to reduce fatigue.

Participants were asked to respond to the target stimuli presented in the first and second displays in both conditions. In each trial, participants had to identify targets in two different compound stimuli. In the changed-level block, when “G” (“L”) was presented as the predictive cue, participants were asked to respond to the global (local) numerals in the first compound stimulus display and to the local (global) numerals in the second display. On the contrary, in the repeated-level block, when “G” (“L”) was presented as a predictive cue, participants responded to the global (local) numerals in both displays. In all trials, participants had to press the left mouse button when the target numeral was 5 or 7 and the right button when the target was 6 or 8.

## 3. Results

### 3.1. Results of IOR on the GPE

All results of the first stimuli for the correct responses were submitted to a 2 × 2 × 2 repeated measures analysis of variance (ANOVA), with condition (changed level vs. repeated level), location (cued vs. noncued), and task (global vs. local) as the within-subject factors. A significance level of 0.05 was used.

The main effects of condition and location were significant (*F*(1, 17) = 26.352, *p* < 0.0005; *F*(1, 17) = 9.345, *p*=0.007). These effects indicated that RTs were slower in the changed-level condition than in the repeated-level condition (1038.82 ms vs. 832.51 ms) and slower for the cued location than for the noncued location (1039.48 ms vs. 1009.94 ms); in other words, we observed IOR. The main effect of task was not significant (*F*(1, 17) = 3.149, *p*=0.094). These main effects were modulated by a significant location × task interaction (*F*(1, 17) = 8.571, *p*=0.009), which indicated that interference was observed only in the cued location (and not in the noncued location).

**Figure 3:**
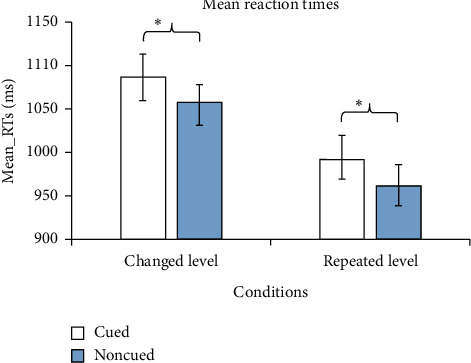
RTs for the cued and the noncued locations in the two conditions.

**Figure 4:**
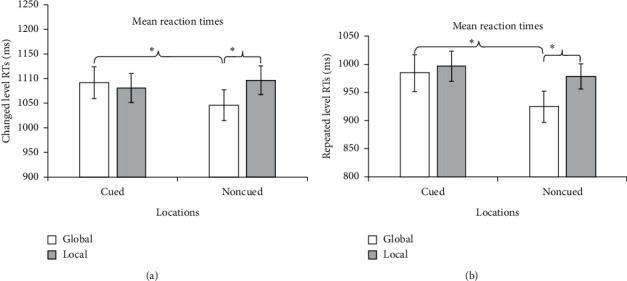
RTs for the experimental tasks. (a) Mean RTs for the global and local tasks at the cued and the noncued locations in the changed-level condition; (b) mean RTs for the global and local tasks at the cued and noncued locations in the repeated-level condition. ^*∗*^*p* < 0.05.

In the changed-level condition, RTs were significantly greater for targets in the cued locations than for targets in the noncued locations (1086.60 ms vs. 1058.30 ms) (*F*(1, 17) = 21.202, *p* < 0.001). In other words, we observed IOR (see Figure 3). When the cue was valid, the main effect of task (global or local) was not significant (1091.48 ms vs. 1080.83 ms) (*F*(1, 17) = 0.174, *p*=0.682; see Figure 4(a)). However, when the cue was invalid, participants' responses for the global tasks were significantly faster than those for the local tasks (1045.94 ms vs. 1096.73 ms) (*F*(1, 17) = 20.113, *p* < 0.001; see Figure 4(a)). The results indicated that the GPE was found in the noncued location but not in the cued location. Moreover, the main effect of cue type in the global conditions was significant (*F*(1, 17) = 11.363, *p*=0.004). However, the main effect of the cue type in the local conditions was not significant (*F*(1, 17) = 0.623, *p* > 0.05). The results showed that the responses to the cued targets were significantly slower only for the global condition. It indicated that IOR affected the processing of inhibited global and local targets, especially the targets at the global level.

In the repeated-level condition, RTs were significantly slower for targets in the cued location than for targets in the noncued location, again indicating IOR (992.35 ms vs. 961.57 ms) (*F*(1, 17) = 16.334, *p* < 0.005; see Figure 3). When the cue was valid, the main effect of task was not significant (984.22 ms vs. 996.25 ms) (*F*(1, 17) = 0.392, *p*=0.540; see Figure 4(b)). However, when the cue was invalid, participants had significantly faster responses in the global tasks than in the local tasks (924.42 ms vs. 978.31 ms) (*F*(1, 17) = 9.183, *p* < 0.01; see Figure 4(b)); that is, we again observed the GPE only in the noncued location. The main effect of the cue type in the global conditions were significant (*F*(1,17) = 12.972, *p*=0.002). However, the main effect of the cue type in the local conditions were not significant (*F*(1, 17) = 2.742, *p*=0.116). The results also showed that the perception to the global features at the cue position was slower than that at the noncue position. Also, the responses to the local tasks in the cued location were not faster than those in the noncued location. It indicated that the disappearance of the GPE on the cued location was because IOR slowed down the cognition on the global features.

### 3.2. Results of Level-Priming on the GPE

**Figure 5:**
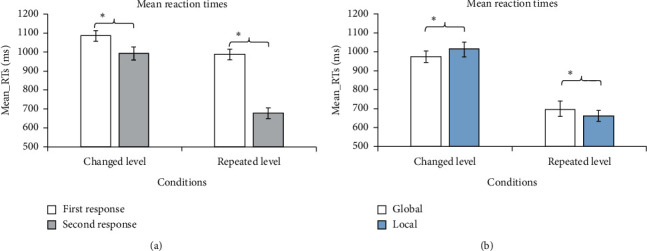
RTs for the experimental tasks. (a) Mean RTs for the first and the second task display responses in the two conditions; (b) mean RTs for the global and local tasks in the two conditions. ^*∗*^*p* < 0.05.

There were obvious differences between the response times of the first stimuli and those of the second stimuli in the changed-level condition (1078.75 ms vs. 992.65 ms) (*F*(1, 17) = 15.184, *p*=0.001), as well as in the repeated-level condition (970.80 ms vs. 677.54 ms) (*F*(1, 17) = 332.353, *p* < 0.0005), as shown in Figure 5(a). The RTs for the condition of the repeated level were faster than those for the condition of the changed level. Specifically, participants tended to be faster in responding to the second display than the first display, regardless of the condition. Notably, the RT difference in the changed-level condition was significantly smaller than that in the repeated-level condition (see Figure 5(a)) (*F*(1, 17) = 141.382, *p* < 0.001). That is, a main effect of level-priming was found in RTs.

All results of the correct responses in the second stimuli were submitted to a 2 × 2 repeated measures analysis of variance (ANOVA), with condition (changed level vs. repeated level) and task (global vs. local) as the within-subject factors. The main effect of condition was significant (*F*(1, 17) = 359.718, *p* < 0.0005). This effect indicated that RTs were slower in the changed-level condition than those in the repeated-level condition for the second stimuli (992.65 ms vs. 677.54 ms). The main effect of task was not significant (*F*(1, 17) = 0.074, *p*=0.787). These main effects were modulated by a significant condition × task interaction (*F*(1, 17) = 10.659, *p*=0.002), which indicated that task interference was observed only for the condition of the repeated level, but not for the condition of the changed level.

In the changed-level condition, RTs were significantly faster for the global tasks than those for the local tasks (975.01 ms vs. 1011.81 ms) (*F*(1, 17) = 7.219, *p*=0.011; see Figure 5(b)). That is, we observed the GPE in the changed level. However, in the repeated-level condition, RTs were significantly slower for the global tasks than those for the local tasks (692.95 ms vs. 660.60 ms) (*F*(1, 17) = 6.145, *p*=0.018; see Figure 5(b)). We not only observed no significant GPE in the repeated-level but also responses for the local tasks were obviously faster than those for the global tasks.

## 4. Discussion

The aim of the present experiment was to study whether IOR and level priming can affect the perception of global and local features in compound stimuli and, furthermore, its impact on the GPE.

We observed that participants showed significantly slower responses to cued locations than to noncued locations. This indicated the presence of IOR. In noncued locations, participants showed faster responses for the global tasks than for the local tasks when they responded to the first stimuli, indicating the presence of the GPE. However, in cued locations, we observed no significant difference in RTs between the global and the local tasks. When the cue was valid, the GPE disappeared. Moreover, the analysis results showed that the RTs of global tasks using cued locations were significantly slower than those using noncued locations, and there was no significant difference in the RTs of local tasks between the cued locations and the noncued locations. It indicated that the reason of disappearance of the GPE was that the inhibitory effect of IOR on the cued location was actually inhibiting the perception of global and local features, and it especially slowed down the perception of global features. Normally, global features can be perceived better than the local features. At the same time, global features were allocated more attentional resources than local features so that the global information can be fully utilized [19, 20]. A previous study showed that the more the attentional resources the participants allocated, the greater the inhibitory effect of IOR [21, 22]. In the experiment, the researchers found that the effect of IOR in the discrimination tasks was significantly greater than that in the detection tasks. The discrimination tasks had more complicated information and took up more attention resources than the detection tasks, thus leading to an increase in the effect of IOR. The magnitude of the effect of IOR depended on the amount of attention resource immediately obtained during information processing. It may be for this reason that IOR had a stronger inhibitory effect on the perception of global features than local features in the stage of inhibitory processing, further causing the disappearance of the GPE.

IOR is a kind of short-term inhibitory effect in cued locations [23–25]. The results of this experiment showed that there were significant inhibitory effects neither in cued locations nor noncued locations when participants responded to the second stimuli. Therefore, IOR does not affect processes of the participants to the second stimuli.

Analysis of RTs to the second stimuli indicated that there was no significant global precedence in the RTs in the repeated-level condition. Not only the GPE disappeared but also the RTs to the global tasks were obviously slower than those to the local tasks. However, the GPE existed in the changed-level condition. Furthermore, we observed a significant level-priming in the condition of the repeated level. Therefore, it was reasonable to conclude that level-priming had an impact on the processing of global and local features, resulting in the disappearance of the GPE. Although level-priming promoted RTs for global and local features, the RTs of the participants to local features were significantly faster than those to global features. We speculated that this was due to the smaller effect of level-priming on global features but the larger effect of level-priming on local features. The phenomenon was in accordance with previous studies that found processing efficiency increased with decreases in the size of the attention area [26]. When the position of the stimulus was fixed, RTs for small letters were significantly shortened, but RTs for large letters was unaffected [27]. In the present study, the second set of stimuli were presented at the same locations as the first set of stimuli in the repeated-level condition, in which the subjects continue to focus their attention to the same location. Thus, level-priming had a greater effect on local stimuli than on global stimuli. Perhaps, another reason for this was spatial frequency. Level-priming is closely related to spatial frequency information [28, 29]. Low spatial frequency is more closely associated with the global level and high spatial frequency is more closely associated with the local level, and the factor providing global advantage was the difference in spatial frequency between the global features and the local features [30, 31]. There was also a significant interaction between the previous level and spatial frequency. Participants were faster when reporting high spatial frequency orientation following the local level than when reporting low spatial frequency orientation following the global level [32]. This supported our hypothesis that a difference in spatial frequency information would result in the difference in the level-priming effect. Therefore, level-priming had more influence on the processing of local features in the repeated-level condition, resulting in the disappearance of the GPE.

## 5. Conclusions

First, we found that IOR had a significant inhibitory effect on the perception of global features and, hence, led to the disappearance of the GPE at cued locations. This suggested that better selection of features utilization in attention processing led to a greater inhibitory effect. Second, we found that level-priming not only accelerated responses of participants to global features and local features but also had a greater promoting effect on the RTs of local features than those of global features.

## Data Availability

The data used to support the findings of the study are included within the article.
